# Revealing genetic causality between blood-based biomarkers and major depression in east Asian ancestry

**DOI:** 10.3389/fpsyt.2024.1424958

**Published:** 2024-09-10

**Authors:** Xiaoxiao Mo, Chao Wang, Qiuyi Pu, Zhengdong Zhang, Dongmei Wu

**Affiliations:** ^1^ Department of Genetic Toxicology, The Key Laboratory of Modern Toxicology of Ministry Education, Center for Global Health, School of Public Health, Nanjing Medical University, Nanjing, China; ^2^ Department of Environmental Genomics, School of Public Health, Nanjing Medical University, Nanjing, China

**Keywords:** major depression, mendelian randomization, basophil, low-density lipoprotein cholesterol, MFN2

## Abstract

**Introduction:**

Major Depression (MD) is a common mental disorder. In East Asian ancestry, the association, causality, and shared genetic basis between blood-based biomarkers and MD remain unclear.

**Methods:**

We investigated the relationships between blood-based biomarkers and MD through a cross-sectional study and Mendelian randomization (MR) analysis. Cross-trait analysis and enrichment analyses were used to highlight the shared genetic determinants and biological pathways. We conducted summary data–based MR to identify shared genes, which were then validated using a transcriptome dataset from drug-naïve patients with MD.

**Results:**

In the cross-sectional study, C-Reactive Protein showed the significantly positive correlation with depressive symptoms, while hematocrit, hemoglobin, and uric acid exhibited significantly negative correlations. In MR analysis, basophil count (BASO) and low-density lipoprotein cholesterol (LDLc) had a significant causal effect on MD. The enrichment analysis indicated a significant role of inflammatory cytokines and oxidative stress. The shared genes *MFN2*, *FAM55C*, *GCC2*, and *SCAPER* were validated, with *MFN2* identified as a pleiotropic gene involved in MD, BASO, and LDLc.

**Discussion:**

This study highlighted that BASO and LDLc have a causal effect on MD in East Asian ancestry. The pathological mechanisms of MD are related not only to inflammatory cytokines and oxidative stress but also to down regulation of *MFN2* expression and mitochondrial dysfunction.

## 1 Introduction

Major Depression (MD) is a common mental disorder, characterized by symptoms like depressed mood or loss of interest and pleasure, persisting for at least two weeks (1). Globally, the prevalence of MD was about 3.15% in 2020, accounting for nearly 50 million disability-adjusted life years (2). In East Asia, the lifetime prevalence of MD is 6.9% with an incidence rate of 2,882.9 per 100,000 people in 2019 (3, 4).

There is growing clinical and preclinical evidence suggesting that the pathogenesis of psychiatric disorders may involve increased peripheral, cerebral inflammation, and oxidative stress (5, 6). Inflammatory mediators released by blood cells, such as cytokines, chemokines, and proteins in the complement system, can reach the brain through various pathways, including neurological, humoral, and chemical routes. These mediators can activate inflammasomes and affect immune cell movement (7, 8). MD is associated with higher plasma levels of proinflammatory cytokines, such as IL-4, IL-5, IL-6, and C-reactive protein (CRP) (9–11). Inflammation and oxidative stress are interconnected and function together (6). In MD, inflammation activates oxidative and nitrosative stress pathways, leading to increased production of reactive oxygen species (ROS) and reactive nitrogen species (RNS) (12). MD is also characterized by decreased antioxidant levels, including low serum concentrations of antioxidants like zinc, copper, tyrosine, and tryptophan (13–15).

Alterations in blood cell function and properties have been linked to various mental and neurological disorders, including MD, schizophrenia (16), multiple sclerosis (17), stroke (18), and Parkinson’s disease (19). Additionally, counts of monocytes, platelets, basophils, and serum uric acid levels have been associated with MD in cross-sectional studies and randomized controlled trials (20–23). It is believed that the associations between blood-based biomarkers and MD share genetic basis (24). Several studies using genome-wide association study (GWAS) summary statistics from individuals of European ancestry have demonstrated relationships between lymphocyte count, monocyte percentage of white cells, and psychiatric disorders. These studies further explored the causality and biological mechanisms between these blood-based biomarkers and MD (24–26). However, the association, causality, and shared genetic basis between blood-based biomarkers and MD remain unclear in individuals of East Asian ancestry.

**Figure 1:**
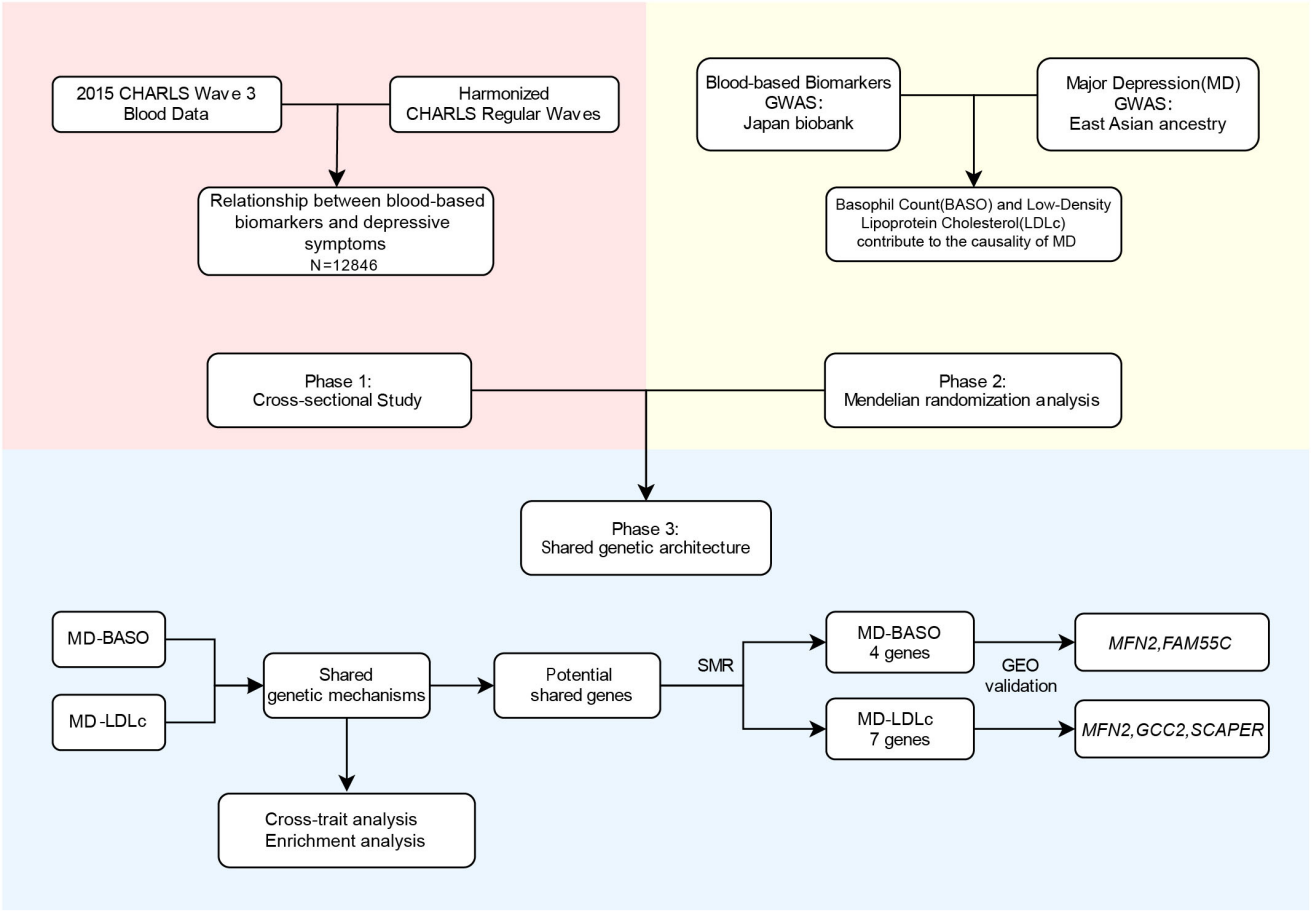
Overview of the main analyses in the study. CHARLS, China Health and Retirement Longitudinal Survey; MD, major depression; BASO, basophil count; LDLc, low-density lipoprotein cholesterol; GEO, Gene Expression Omnibus dataset.

The aim of this study is to investigate the association and causality between blood-based biomarkers and MD in individuals of East Asian ancestry and to comprehensively characterize the shared genetic basis and biological mechanisms. First, we conducted a cross-sectional study to investigate the association between blood-based biomarkers and MD, based on the China Health and Retirement Longitudinal Study (CHARLS, wave 3). Subsequently, MR analysis was performed to examine the causality between blood-based biomarkers and MD using GWAS summary statistics. Furthermore, single-cell, and pathway enrichment analyses were conducted to explore the biological mechanisms. Finally, shared genes between blood-based biomarkers and MD were identified using summary data–based MR (SMR). The transcriptome study from Gene Expression Omnibus (GEO) database was used to determine whether the shared genes were differentially expressed between patients and controls. The detailed study design is shown in **Figure 1**.

## 2 Methods

### 2.1 Data collection

We conducted a cross-sectional study based on the CHARLS, which aimed to collect a high-quality, nationally representative sample of Chinese residents aged 45 and older. The baseline national wave of CHARLS commenced in 2011, approximately 17,500 individuals. As for our study, data from wave 3 was utilized because it contained extensive blood-based bioassays. This study utilized CHARLS data, comprising datasets from the 2015 CHARLS Wave 3 Blood Data (Version ID: 20190620) and Harmonized CHARLS Regular Waves (Version as of June 2021). All analyses in this study adhered to the Data Release Note (27).

### 2.2 Measurement of depressive symptoms

The Center for Epidemiologic Studies Depression Scale 10 (CES-D10) comprises questions posed on a 4-point scale, corresponding to the frequency of feelings experienced by individuals over the past week. The CES-D10 score ranges from 0 to 30. The reliability and validity of the CES-D10 have been previously established (28). Data from the CES-D10 were available for 25,586 participants. Depressive symptoms were transformed into categorical variable based on the CES-D10 score. With reference to previous studies, a cut-off score of 12 was valid for identifying clinically depression (29, 30). Cutoff scores for depressive symptoms were set at 12 for the 10-item questionnaire. Therefore, if the score is 12 or higher, the individual had depressive symptoms.

### 2.3 Blood-based bioassays

The Blood-Based Bioassays encompassed a total of 16 blood-based biomarkers, all of which were continuous variables (**Supplementary Table 1**). The Blood-Based Bioassays samples were assayed for high-sensitivity CRP, glycated hemoglobin (HbA1c), total cholesterol (TCHO), HDL cholesterol (HDL-C), LDL cholesterol (LDL-C, referred to as LDLc), triglycerides (TG), glucose (BG), blood urea nitrogen (BUN), creatinine (SCr), uric acid (UA), and cystatin C (CysC). Additionally, blood count analyses were included comprising hemoglobin (HGB), hematocrit (HCT), white blood cell count (WBC), platelet counts (PLT), and mean corpuscular volume (MCV). Blood data was available for 13,420 participants.

### 2.4 Covariates

Covariate variables were obtained from the Harmonized CHARLS datasets, which were categorized into quantitative variables (age and economic situation) and qualitative variables (gender, smoking, alcohol consumption, and education level) (**Supplementary Table 1**). Notably, the education level was determined based on the integration of raw data. The variable RAEDUC_C was defined using the following codes: 1. No Formal Education (Illiterate), 2. Did Not Finish Primary School but can Read, 3. Sishu (Private Tutoring), 4. Elementary School, 5. Middle School, 6. High School, 7. Vocational School, 8. Two/three-year college, 9. College Grad and 10. Post-graduate degree. We recoded levels 1-3 as 1 (labeled as “Before Elementary School”), level 4 as 2 (labeled as “Elementary School”), level 5 as 3 (labeled as “Middle School”), levels 6-7 as 4 (labeled as “High School”), and levels 8-10 as 5 (labeled as “Above Three-year College”).

### 2.5 Missing data

First, individual IDs were used to merge the blood bioassays data and the harmonized CHARLS dataset. The missing data were categorized into three types: basic demographic characteristics, blood-based biometric indicators, and CES-D10 scores. For the first type of missing data, if the missing rate was not more than 2%, the samples containing missing data were excluded. For the variable of economic situation, which had a missing rate of about 15%, the missing data was excluded in Model 3. For the second type of missing data, multiple imputation by chained equations (MICE) was performed based on random forests (31, 32). For the third type, if there were missing answers but the CES-D 10 score was greater than or equal to 12, or there is no missing data, the individual would be included in the analysis.

### 2.6 Multi-omics data sources

We used the GWAS summary statistics of 23 blood-based biomarkers (33) and MD (34) in East Asian ancestry (**Figure 1**, **Supplementary Table 1**). The 23 blood-based biomarkers GWAS were all sourced from the Japan Biobank, which collected DNA and serum samples from 12 medical institutions in Japan and recruited approximately 200,000 participants. MD GWAS data was sourced from Giannakopoulou et al. including 15,771 individuals with depression and 178,777 control participants from 9 different studies. In SMR analysis, we utilized expression quantitative trait loci (eQTL) datasets from Japanese (35). Additionally, we obtained genome-wide profiling of the transcriptome in the peripheral blood of drug-naïve MD patients and health controls (GSE201332) (36). The single-cell RNA sequencing (scRNA-seq) data from peripheral blood (GSE112845) was downloaded from ABC portal (http://abc.sklehabc.com) (37, 38).

### 2.7 Statistical analysis

#### 2.7.1 Observational analysis

In this study, we estimated the correlation of blood-based biomarkers on depressive symptoms using logistic regression model analysis. We selected blood-based biomarkers with statistically significant results (*P*<0.05) from the univariate analysis to further perform multivariate regression. In Model 1, we adjusted for age and gender. Model 2 additionally adjusted for smoking and alcohol consumption based on Model 1. Model 3 further adjusted for education level and economic situation based on Model 2. We assessed the statistical significance of the effects of variables on the outcome of depressive symptoms using odds ratio (OR), 95% confidence intervals (95%CI), and *P* value of Model 3. Using a false discovery rate (FDR) threshold of 5% for multiple testing correction.

#### 2.7.2 MR analysis

We used MR analysis to estimate the magnitude of the causal relationship between 23 blood-based biomarkers and MD. MR is a method that uses genetic variants as instrumental variables (IVs) to infer causality (39). The Inverse Variance Weighted method (IVW) was utilized as the main analysis using a random-effects model (40). Additionally, 6 alternative MR methods (CAUSE, GSMR, MR-Egger, MR-PRESSO, Weighted Median, and Weighted Mode) were employed as sensitivity analyses to reinforce the results (41–44). Valid IVs need to satisfy the assumption that IVs are strongly associated with the exposure, but not directly associated with the outcome or confounding factors. We selected independent significant SNPs as IVs (*P*<5×10^-8^, physical distance >10,000 kb, and LD r^2^<0.001 base on 1000 Genomes Project phase 3 of East Asian population). Then, IVs were removed if they were significantly (*P*<5×10^-8^) associated with the outcome. We categorized the 23 blood cell phenotypes into three groups: metabolic category, blood cell, and inflammatory/kidney-related category. Based on different categories of blood-based biomarkers, the FDR threshold of 5% was utilized to correct the results of IVW method to reduce false positives. Additionally, if there is a single IV, the Wald ratio is used as the main analysis and CAUSE as the sensitivity analysis (45). Horizontal pleiotropy test and Cochran’s heterogeneity test were performed as sensitivity analyses.

#### 2.7.3 The enrichment analyses

A cross-trait analysis using Pleiotropic Analysis under Composite null hypothesis (PLACO) was conducted to identify shared genetic components between MD and BASO as well as LDLc (46). Unlike conventional cross-trait analyses that test the global null hypothesis that there is no association of a genetic variant with any of the traits, PLACO test a composite null hypothesis of no pleiotropy is that at most one trait is associated with the genetic variant. Thus, the alternative hypothesis suggests that two traits is significantly associated with the genetic variant.

For significant pleiotropic variants identified by PLACO, we employed gene level analysis by Multi-marker Analysis of Genomic Annotation (MAGMA v1.10). Then, we used Functional Mapping and Annotation (FUMA v1.5.2) for pathway enrichment analysis (47). Initially, Gene Ontology (GO) gene sets from MsigDB v2023.1. Hs were utilized for pathway enrichment analysis (48). We perform a pathway-based polygenic regression method (scPagwas) to discover trait-relevant cells (49, 50).

#### 2.7.4 SMR

SMR, an analysis similar to the MR framework, combines GWAS summary statistics with eQTL datasets to identify genes associated with complex traits. Heterogeneity in dependent instruments (HEIDI) uses multiple SNPs in a cis-eQTL region to distinguish pleiotropy from linkage. The SMR & HEIDI methodology can be interpreted as an analysis to test if the effect size of a SNP on the phenotype is mediated by gene expression (51). Significant genes are defined as genes with *P*_SMR_<0.05 and *P*_HEIDI_>0.01.

### 2.8 Database validation

To validate potential shared genes, we utilized the published transcriptome study of whole blood samples from patients with depression in Chinese population (36). The study sample included 20 healthy controls and 20 drug-naïve MD patients. Genes that survived multiple test corrections were defined as significant genes.

## 3 Results

### 3.1 Relationship between blood-based biomarkers and depressive symptoms

A total of 12,846 samples were analyzed after excluding those with missing data and conducting multiple imputations. The basic demographic characteristics, blood-based biomarkers, and CES-D10 scores of the participants are presented in **Supplementary Tables 1** and **2**. The mean CES-D10 score for all participants was 8.0 ± 6.3, with 3,310 participants identified as depressive symptoms. Univariate logistic regression results showed the significant differences in CRP, CysC, HCT, HDL-C, HGB, MCV, SCr, and UA between individuals with depressive symptoms and controls (**Supplementary Table 3**). In the multivariate analysis, CRP was significantly positively correlated with depressive symptoms in Model 3 (OR=1.011, 95% CI=1.004-1.018, *P*_adjust_ = 0.005) after multiple testing correction. HCT and UA showed significant negative correlations with depressive symptoms across all three adjusted models (**Table 1**). There were significant positive correlations between CysC and HGB and depressive symptoms in Model 1 and Model 2, but not in Model 3 after multiple testing correction. No significant association was found between HDL-C, MCV, SCr and depressive symptoms.

**Table 1:** Multivariable analysis in CHARLS between blood-based biomarkers and MD.

	Models	*β*	SE	OR	OR_95%low	OR_95%up	*P*	*Padjust*
CRP	1	0.012	0.003	1.012	1.005	1.018	**<0.001**	**0.001**
	2	0.012	0.003	1.012	1.005	1.018	**<0.001**	**0.001**
	3	0.011	0.004	1.011	1.004	1.018	**0.003**	**0.005**
CysC	1	0.298	0.091	1.347	1.129	1.612	**0.001**	**0.003**
	2	0.281	0.091	1.324	1.109	1.584	**0.002**	**0.004**
	3	0.199	0.103	1.221	0.997	1.495	0.053	0.084
HCT	1	-0.020	0.004	0.981	0.973	0.988	**<0.001**	**<0.001**
	2	-0.020	0.004	0.980	0.972	0.988	**<0.001**	**<0.001**
	3	-0.014	0.005	0.986	0.977	0.994	**0.001**	**0.003**
HDL-C	1	0.003	0.002	1.003	0.999	1.006	0.162	0.229
	2	0.003	0.002	1.003	0.999	1.007	0.097	0.145
	3	0.001	0.002	1.001	0.997	1.005	0.659	0.832
HGB	1	-0.042	0.012	0.959	0.937	0.982	**0.001**	**0.002**
	2	-0.043	0.012	0.958	0.936	0.981	**<0.001**	**0.001**
	3	-0.028	0.013	0.972	0.947	0.998	**0.034**	0.059
MCV	1	0.000	0.003	1.000	0.994	1.005	0.870	0.994
	2	-0.001	0.003	0.999	0.994	1.004	0.757	0.908
	3	-0.001	0.003	0.999	0.993	1.004	0.622	0.829
SCr	1	0.000	0.077	1.000	0.853	1.158	0.995	0.995
	2	0.001	0.077	1.001	0.854	1.159	0.985	0.995
	3	-0.004	0.081	0.996	0.842	1.161	0.956	0.995
UA	1	-0.069	0.016	0.934	0.904	0.964	**<0.001**	**<0.001**
	2	-0.066	0.016	0.936	0.907	0.967	**<0.001**	**<0.001**
	3	-0.058	0.018	0.944	0.910	0.978	**<0.001**	**0.004**

SE, standard error; *Padjust*, using a false discovery rate (FDR) threshold of 5%. Bold values indicate statistically significant p-values.

### 3.2 Genetic causality between blood-based biomarkers and MD

**Figure 2:**
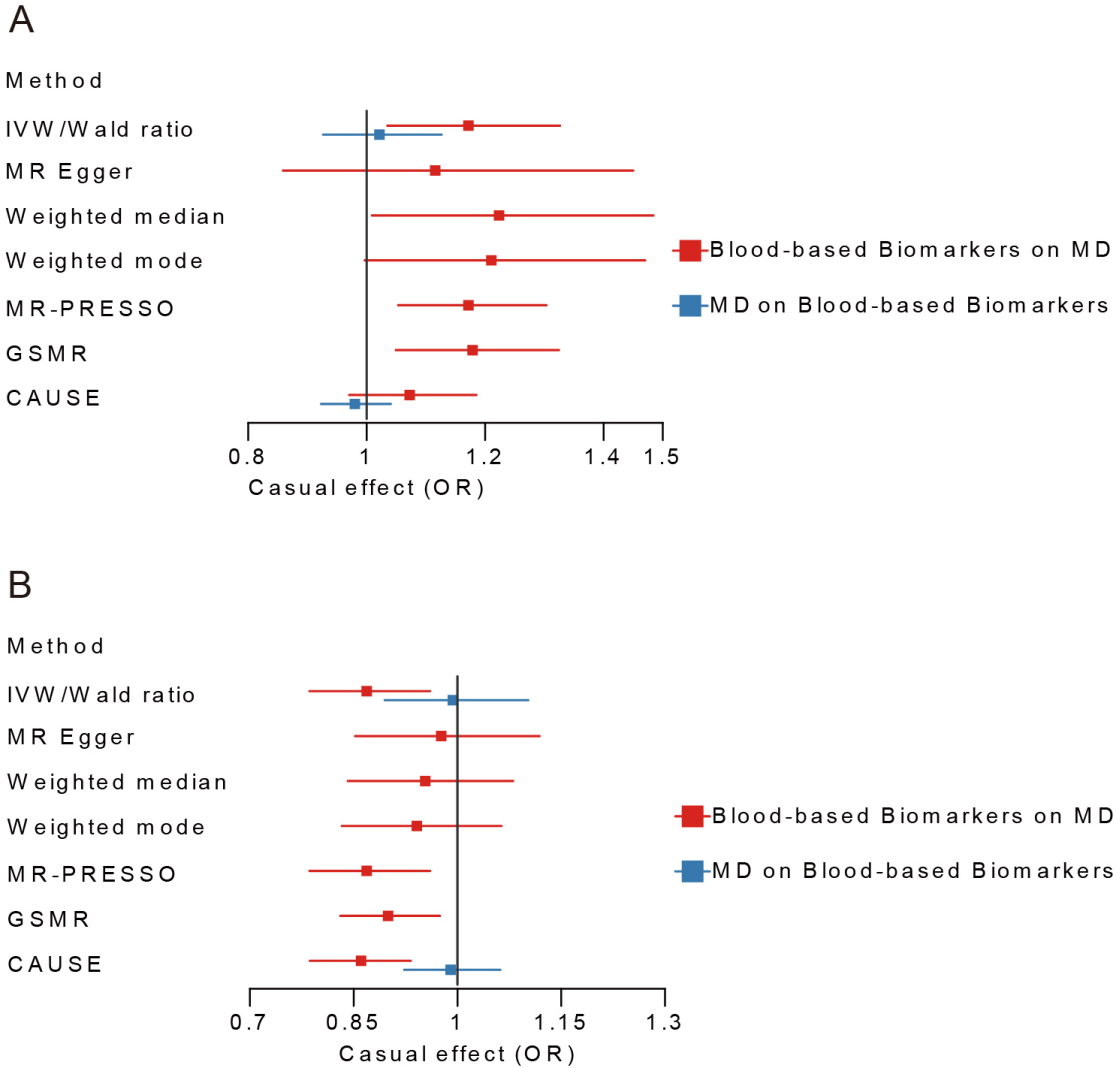
Bi-directional Mendelian Randomization analyses between BASO, LDLc and MD. **(A)** MD-BASO. **(B)** MD-LDLc. Blue color represents the estimated causal effect of MD on blood-based biomarkers, red represents the estimated causal effect of blood-based biomarkers on MD. MD, major depression; BASO, basophil count; LDLc, low-density lipoprotein cholesterol; IVW, Inverse Variance Weighted; CAUSE, Causal Analysis Using Summary Effect Estimates; GSMR, Generalized Summary-data-based Mendelian Randomization.

In blood cell category, BASO had a suggestive causal effect on MD (OR=1.17, 95% CI=1.04-1.33, *P*=0.012), with 3 out of 6 alternative MR analyses supporting this result. In metabolic category, LDLc showed significantly causal effects on MD (OR = 0.87, 95% CI=0.79-0.96, *P*=0.006), with 3 out of 6 alternative MR analyses in consistent with this finding (**Figure 2**; **Supplementary Tables 4**, **5**). It was worth noting that this causal effect was still significant after multiple testing correction. The sensitivity analysis showed no heterogeneity of IVs both in BASO, LDLc, and MD (**Supplementary Tables 6**, **7**). In Inflammatory/Kidney-related category, no causal effect between other blood-based biomarkers and MD was found (**Supplementary Table 4**). The trait pairs of BASO and LDLc with MD were included in subsequent analyses due to the priority of MR in causal inference.

### 3.3 Pathway enrichment and single-cell analyses

**Figure 3:**
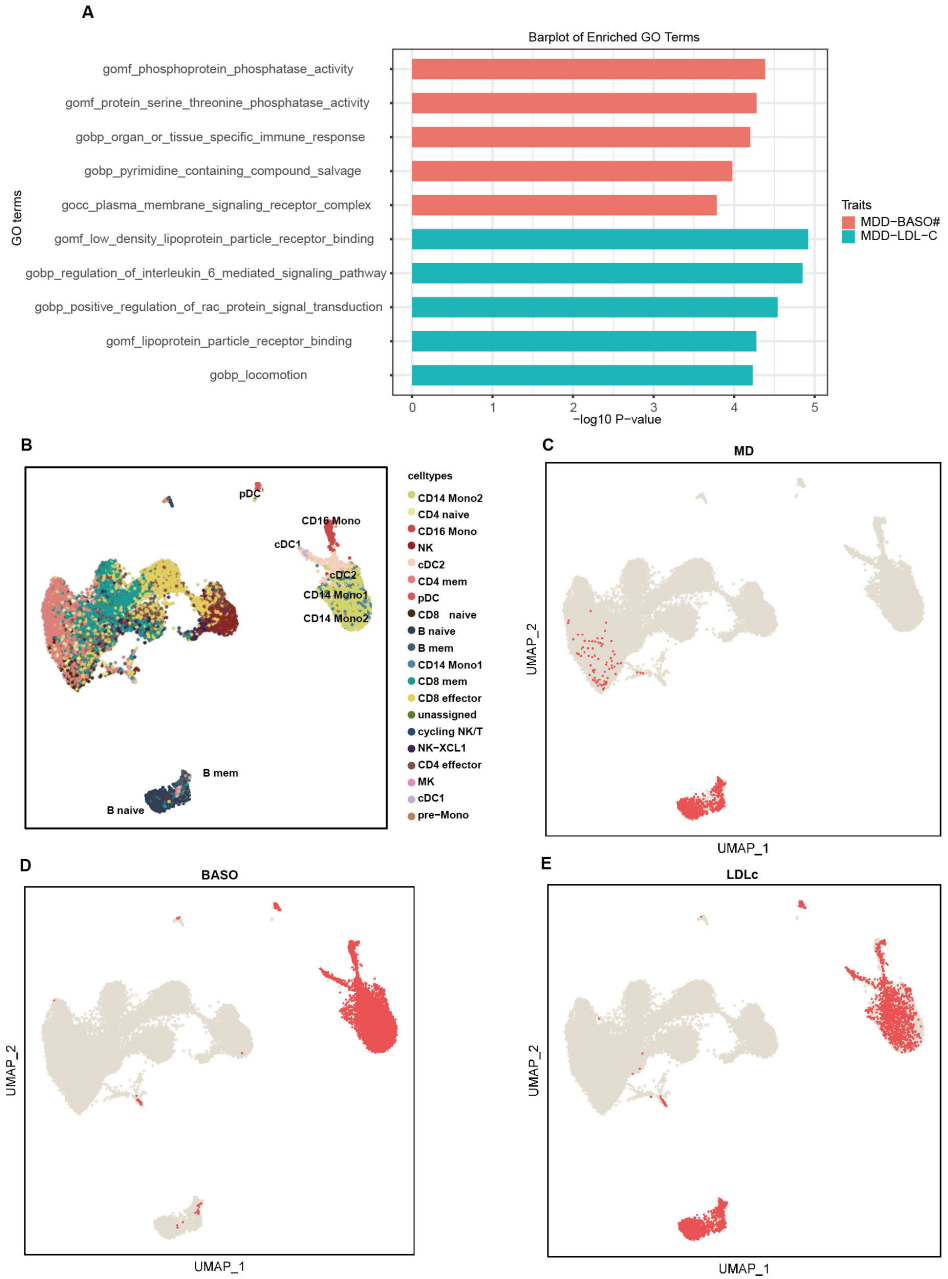
The pathway and single-cell enrichment analysis. **(A)** The pathway enrichment analysis. The x-axis represents -log10 P-values, and the y-axis represents top 5 gene sets across datasets. **(B)** The umap plot shows the cell type labels. **(C–E)** Per-cell TRSs after background correction calculated by scPagwas (Seurat) for three traits including MD **(C)**, BASO **(D)**, and LDLc **(E)** are shown in per cell type.

Based on PLACO, gene set enrichment analysis identified 800 nominally significant pathways associated with the MD-BASO trait pair, but none remained significant after Bonferroni correction. The top five pathways mainly involved phosphatase activity and immune response (**Figure 3A**). Notably, phosphoprotein phosphatase activity (GO:0004721) and protein serine/threonine phosphatase activity (GO:0004722), which regulate the phosphorylation state of cellular proteins, were highlighted. Additionally, the pathway specific to organ or tissue immune response (GO:0002251) was significantly enriched.

For the MD-LDLc trait pair, 840 pathways were significantly enriched, but none remained significant after Bonferroni correction. The top five pathways were primarily related to low-density lipoprotein receptors, cytokine regulation, and locomotion (GO:0040011) (**Figure 3A**). Moreover, pathways related to inflammation, such as IL-6 (GO:0070102) and RAC protein (GO:0035022), were also emphasized.

scPagwas identified that B cells (naive and memory B cells) were associated with MD, and dendritic cells (cDC1 and cDC2) along with mononuclear cells (CD16 mono, CD14mono1, and CD14mono2) were associated with BASO (**Figures 3B–D**). Interestingly, all of these cell types were also related to LDLc (**Figure 3E**).

### 3.4 Share genes identification and transcriptome validation

**Figure 4:**
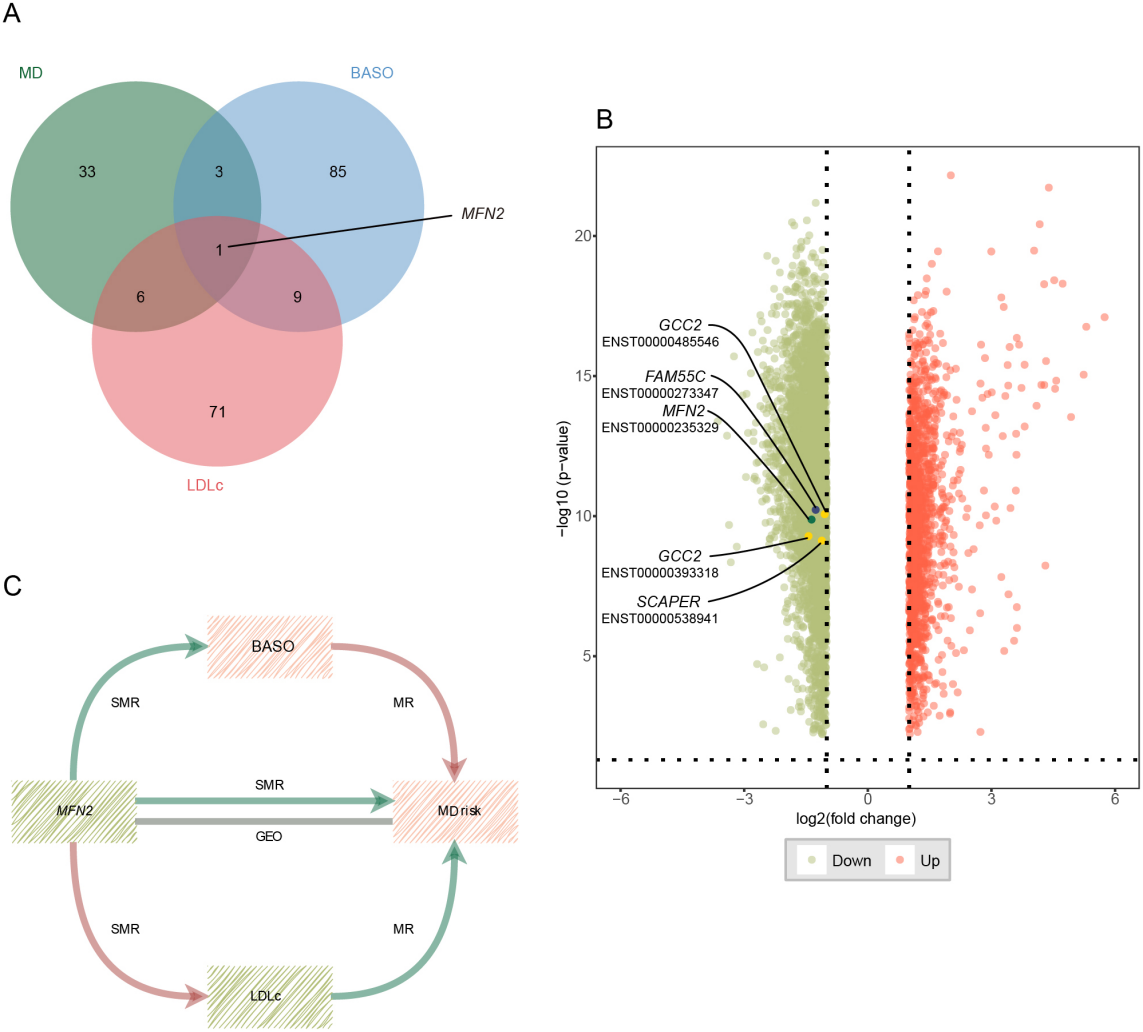
Potential shared genes identification and validation. **(A)** The Venn diagram of shared genes identified by SMR across three traits. **(B)** Volcano plot for differential expression analysis between MD patients and health control. Vertical and horizontal dashed lines represent the thresholds for discovering differentially expressed genes. Log2 fold change >1 and *P*<0.01. Red color represents up-regulated genes, and green color represents down-regulated genes. The points with the name and transcript are validated genes. **(C)** The critical role of *MFN2* between BASO, LDLc and MD. Red color represents up-regulation or positive correlation, and green color represents down-regulation or negative correlation. MD, major depression; BASO, basophil count; LDLc, low-density lipoprotein cholesterol.

We identified 43 nominally significant genes for MD, 98 for BASO, and 87 for LDLc (**Figure 4A**). The MD-BASO trait pair had four shared genes (*MFN2*, *AC074286.1*, *FAM55C*, and *RP11-115L11.1*), while the MD-LDLc trait pair had seven shared genes (*MFN2*, *GCC2*, *GPR20*, *CTD-3064M3.3*, *CKAP2*, *SCAPER*, and *RPL36P4*). Notably, one pleiotropic gene was identified among BASO, LDLc, and MD (**Figure 4A**). Furthermore, the expression of *MFN2* (mitofusin 2), *FAM55C* (neurexophilin and PC-esterase domain family member 3), *GCC2* (GRIP and coiled-coil domain containing 2), and *SCAPER* (S-phase cyclin A associated protein in the ER) was down-regulated in GEO, consistent with the SMR results. (**Figure 4B**; **Table 2**). As shown in **Figure 4C**, the down-regulation of MFN2 was significantly associated with an increased risk of MD, as confirmed by differential gene expression results. Additionally, down-regulation of MFN2 was significantly associated with an increase in BASO and a positive causal relationship between BASO and MD. Concurrently, down-regulation of MFN2 was significantly associated with a decrease in LDLc and a causal relationship between LDLc and MD.

**Table 2:** Shared genes between blood-based biomarkers and MD.

Trait pair	Gene	phen	*β*_SMR	se_SMR	*P*_SMR	*P*_HEIDI	nsnp_HEIDI
MD-BASO	*MFN2* ^a^	MD	-0.027	0.013	0.045	0.926	19
		BASO	-0.017	0.004	<0.001	0.344	17
MD-BASO	*AC074286.1*	MD	0.050	0.022	0.023	0.893	10
		BASO	0.016	0.006	0.012	0.750	10
MD-BASO	*FAM55C ^a^*	MD	-0.043	0.019	0.023	0.038	8
		BASO	-0.013	0.005	0.017	0.438	8
MD-BASO	*RP11-115L11.1*	MD	-0.040	0.014	0.006	0.815	13
		BASO	-0.011	0.004	0.008	0.828	12
MD-LDLc	*MFN2 ^a^*	MD	-0.027	0.013	0.045	0.926	19
		LDLc	0.009	0.004	0.029	0.900	17
MD-LDLc	*GCC2 ^a^*	MD	0.098	0.041	0.015	0.011	20
		LDLc	0.013	0.007	0.048	0.016	20
MD-LDLc	*GPR20*	MD	0.045	0.020	0.027	0.755	16
		LDLc	0.016	0.006	0.013	0.023	13
MD-LDLc	*CTD-3064M3.3*	MD	0.035	0.016	0.028	0.833	17
		LDLc	0.013	0.005	0.011	0.273	14
MD-LDLc	*CKAP2*	MD	-0.046	0.017	0.006	0.749	9
		LDLc	-0.012	0.005	0.022	0.227	7
MD-LDLc	*SCAPER ^a^*	MD	-0.045	0.020	0.020	0.658	7
		LDLc	-0.014	0.006	0.019	0.832	6
MD-LDLc	*RPL36P4*	MD	-0.035	0.016	0.028	0.467	6
		LDLc	-0.016	0.006	0.005	0.959	6

MD, major depression; BASO, basophil count; LDLc, low-density lipoprotein cholesterol; SMR, summary data–based Mendelian randomization; HEIDI, heterogeneity in dependent instrument; phen, phenotype; se_SMR, standard error of SMR; nsnp_HEIDI, number of SNPs in the HEIDI test.

a validated by Gene Expression Omnibus dataset.

## 4 Discussion

In this study, we used a combination of cross-sectional and MR analyses to explore the association and causality between blood-based biomarkers and MD. We identified a total of four shared genes (*MFN2*, *AC074286.1*, *FAM55C*, and *RP11-115L11.1*), which *MFN2* being a pleiotropic gene linked to MD, BASO, and LDLc.

In our cross-sectional study, we found associations between depressive symptoms and several blood-based biomarkers, including CRP, HCT, HGB, and UA. CRP showed a significant positive correlation with depressive symptoms, while HCT, HGB, and UA exhibited significant negative correlations. Similarly, previous clinical trials have reported associations between these biomarkers and depressive symptoms. Haapakoski et al. observed higher mean levels of CRP in patients with MD compared to non-depressed controls (10). Lee et al. reported that Korean adults with MD typically have lower HCT levels (52). Additionally, Kesebir et al. found that UA levels were lower in patients with MD than in healthy controls (53). These findings are consistent with our results. However, Lee et al. reported no association between HGB level and MD in Korean adults (52), which differ from our finding that HGB was negative related with depressive symptoms. Several factors might explain this discrepancy. First, our study did not account for family history and stress as confounding factors, which are known genetic and environmental risk factors for depression. The CHARLS cohort aims to describe the basic information of middle-aged and elderly people in China comprehensively, so the questionnaire did not include psychiatric-specific information. Second, the CES-D10 score used to define depressive symptoms is not a standard for the clinical diagnosis of depression. Third, differences in the age and ethnic groups of the participants might have influenced the associations. Unlike the study in Korean adults (52), findings from other studies in older populations were consistent with our results. Trevisan et al. revealed that low HGB strongly predicted incident depression in older Italian men (54). Vulser et al. reported that depression and antidepressant use were associated with lower HGB levels in elderly French individuals (55).

In the MR analysis, BASO was found to have a significant causal effect on MD. Although BASO was not available in the Blood-Based Bioassays and there was no significant association found between WBC and depressive symptoms, the change in basophil count is challenging to detect through WBC because basophils make up less than 1% of circulating leukocytes. However, several cross-sectional and prospective cohort studies have demonstrated a relationship between BASO count and MD. Bai et al. reported that MD patients had significantly increased basophil counts (*P*=0.030) (56). Puangsri et al. found that basophil percentages significantly decreased after antidepressant treatment (*P*=0.027) (23). Although the association between LDLc and depressive symptoms was not significant in our cross-sectional results, MR analysis indicated that LDLc had a causal effect on MD. Previous studies support the idea that alterations in lipid levels may be linked to MD. For example, Aijänseppä et al. found an independent association between low LDLc concentration and depressive symptoms in elderly Finnish men (57). Additionally, a randomized controlled trial suggested that basophil counts predicted cognitive dysfunction in depression when stratified by BMI status, with LDLc also associated with BMI (58, 59). Therefore, further research is needed to explore the shared genetic basis between BASO, LDLc, and MD.

The results of the enrichment analyses supported the hypothesis that inflammation and oxidative stress play roles in the etiology of MD. In pathway enrichment analysis, the pathways GO:0004721 and GO:0004722, related to protein serine threonine/phosphatase activity, were highlighted. Previous studies have shown that two distinct lineages of this phosphatase, the PPP family and the PPM family, can influence depressive-like behaviors by regulating stress pathways, such as by modulating the synthesis of synaptic proteins or neuronal excitability (60, 61). Protein serine/threonine phosphatase have also been shown to affect histamine release from basophils in several studies (62, 63). The pathways GO:0002251 and GO:0070102 were associated with immune response and changes in cytokine levels, such as IL-6. Basophils are involved in the immune response as granulocytes, and the immune dysregulation has been observed in patients with depression (64–66). The shared pathways in the MD-LDLc trait pair emphasized the role of the LDLc receptor and GTPases Rac proteins. Several studies suggest that LDLc may affect stress disorders and depression through the epigenetic regulation of RAC1 (67–70).

We found that both naive and memory B cells were associated with MD and LDLc according to the results of single-cell enrichment analysis. Altered B-cell activation has been reported in association with postpartum depression (71). Furthermore, Diana et al. reported a reduction in naive B cell frequencies in severely depressed patients (72). The apolipoprotein-mediated pathway of lipid antigen presentation in B cells plays a crucial role in the innate help provided by NKT cells (73). IgM antibodies produced by B cells can bind to oxidized low-density lipoprotein, exerting anti-atherosclerotic effects (74). Atherosclerosis has also been reported to associated with MD (75). Therefore, the relationship between LDLc and MD may be mediated through its interaction with B cells.

In this study, the expression of four significantly shared genes were found to be downregulated in MD patients. Notably, *MFN2*, a key player in mitochondrial function, was identified as a gene associated with MD, BASO, and LDLc. *MFN2* may be play a crucial role in mediating the relationship between increased BASO, decreased LDLc and the elevated risk of MD. Mitochondrial dysfunction and increased mitochondrial fragmentation have been observed in *Mfn2* conditional knockout mice, which is associated with appropriate mitochondrial shape, function, and intracellular distribution (76–78). Evidence of mitochondrial damage in MD has been documented in various areas, including inflammation, oxidative stress, and changes in neuroplasticity (79). Animal studies have demonstrated that *Mfn2* in the nucleus accumbens can regulate depressive-like behaviors by affecting mitochondrial structure and function (80). This suggests that *MFN2* is involved in the pathophysiological processes of MD. Furthermore, MFN2 protein levels were found to be downregulated in MD patients, but were restored following treatment with a selective serotonin reuptake inhibitor (81). However, another clinical trial showed increased mitochondrial fragmentation and upregulated MFN2 protein levels in MD patients (82). These conflicting results on MFN2 protein levels in MD patients may arise from discrepancies between changes in *MFN2* gene expression and protein levels.

This study has several limitations. First, the relationship between MD and BASO, as well as LDLc, has not been validated in prospective cohort studies or clinical trials. Second, although the link between *MFN2* and MD has been validated by external databases and supported by prior research, the roles of BASO and LDLc in this relationship remain unclear.

This study research that BASO and LDLc have a causal effect on MD in individuals of East Asian ancestry. These findings suggest that the genetic structures linking BASO, LDLc and MD are related not only to inflammatory cytokines and oxidative stress but also to mitochondrial dysfunction. It is speculated that the down regulation of *MFN2* may play a significant role in the mitochondrial theory of depression.

## Data Availability

The original contributions presented in the study are included in the article/**Supplementary Material**. Further inquiries can be directed to the corresponding author.
