# Supplementary material for: Revealing genetic causality between blood-based biomarkers and major depression in east Asian ancestry
Source: Front Psychiatry. 2024 Sep 10;15:1424958. doi: 10.3389/fpsyt.2024.1424958 (PMC11423294; doi:10.3389/fpsyt.2024.1424958)
Supplement: Supplementary file 1 [file DataSheet1.docx]

Supplementary Materials

**Revealing Genetic Causality Between Blood-Based Biomarkers and Major Depression in East Asian Ancestry**

# Supplementary Figures and Tables

## Supplementary Tables

**Supplementary Table 1. Abbreviation and description for unified variables**

| **Variable1** | **Variable2** | **Characteristics** | **Label** | **Category** | **Unit/Type** |
| --- | --- | --- | --- | --- | --- |
| bl_glu | GLU | BG | Glucose | Metabolic | mg/dl |
| bl_bun | BUN | BUN | Blood Urea Nitrogen (BUN) | Kidney-related | mg/dl |
| bl_crp | CRP | CRP | C-Reactive Protein (CRP) | Inflammatory | mg/l |
| bl_cysc | / | CysC | Cystatin C | / | mg/l |
| bl_hbalc | HBA | HbA1c | Glycated Hemoglobin | Metabolic | % |
| bl_hct | Ht | HCT | Hematocrit | Blood cell | % |
| bl_hdl | HDL-C | HDL-C | High-Density Lipoprotein Cholesterol | Metabolic | mg/dl |
| bl_hgb | Hb | HGB | Hemoglobin | Blood cell | g/dl |
| bl_ldl | LDLC | LDLc | Low-Density Lipoprotein Cholesterol | Metabolic | mg/dl |
| bl_mcv | MCV | MCV | Mean Corpuscular Volume | Blood cell | fl |
| bl_plt | PLT | PLT | Platelets | Blood cell | 10^9/L |
| bl_crea | SCR | SCr | Creatinine | Kidney-related | mg/dl |
| bl_cho | TC | TCHO | Total Cholesterol | Metabolic | mg/dl |
| bl_tg | TG | TG | Triglycerides | Metabolic | mg/dl |
| bl_ua | UA | UA | Uric Acid | Kidney-related | mg/dl |
| bl_wbc | WBC | WBC | White Blood Cell | Blood cell | 1000 |
| / | BAS | BASO | Basophil count | Blood cell | / |
| / | EOS | EO | Eosinophil count | Blood cell | / |
| / | LYM | LYMPH | Lymphocyte count | Blood cell | / |
| / | MCH | MCH | Mean corpuscular hemoglobin | Blood cell | / |
| / | MCHC | MCHC | Mean corpuscular hemoglobin concentration | Blood cell | / |
| / | MON | MONO | Monocyte count | Blood cell | / |
| / | NEU | NEUT | Neutrophil count | Blood cell | / |
| / | RBC | RBC | Red blood cell count | Blood cell | / |
| R3CESD10 | / | CES-D10 score | the sum of 8 negative questions, after reverse coding 2 positive questions | / | continuous |
| R3AGEY | / | Age | the respondent’s age in years at the time of the 3rd wave’s interview | / | continuous |
| RAGENDER | / | Gender | the respondent’s gender | / | categorical |
| R3SMOKEN | / | Smoking | the respondent's current smoking habit | / | categorical |
| R3DRINKL | / | Alcohol consumption | whether the respondent has had an alcoholic beverage in the last 12 months | / | categorical |
| H3ATOTFA | / | Economic situation | the net value of non-housing financial wealth at the couple level | / | continuous |
| RAEDUC_C | / | Education level | the highest level of education that the respondent has attained |  | categorical |

Variable1, cross-sectional study variables; Variable2, genetics study variables; Characteristics, standard abbreviation for variables.

**Supplementary Table 2. Baseline characteristics of CHARLS study sample**

| **Characteristics** |  | **N** | **%** |
| --- | --- | --- | --- |
| BG |  | 103.3 ± 34.9 |  |
| BUN |  | 15.4 ± 4.7 |  |
| CRP |  | 2.7 ± 5.9 |  |
| CysC |  | 0.8 ± 0.2 |  |
| HbA1c |  | 6.0 ± 1.0 |  |
| HCT |  | 41.5 ± 5.7 |  |
| HDL-C |  | 51.2 ± 11.5 |  |
| HGB |  | 13.7 ± 2.0 |  |
| LDLc |  | 102.2 ± 29.0 |  |
| MCV |  | 91.3 ± 7.8 |  |
| PLT |  | 205.1 ± 75.6 |  |
| SCr |  | 0.8 ± 0.3 |  |
| TCHO |  | 183.8 ± 36.6 |  |
| TG |  | 143.2 ± 91.3 |  |
| UA |  | 4.9 ± 1.4 |  |
| WBC |  | 6.0 ± 2.1 |  |
| CES-D10 score |  | 8.0 ± 6.3 |  |
| Depressive symptoms | None | 9536 | 74.20 |
|  | Yes | 3310 | 25.80 |
| Age |  | 59.3 ± 10.0 |  |
| Economic situation |  | 14850.9 ± 271840.0 |  |
| Gender | Male | 5937 | 46.20 |
|  | Female | 6909 | 53.80 |
| Alcohol consumption | None | 8317 | 64.70 |
|  | Yes | 4529 | 35.30 |
| Smoking | None | 9304 | 72.40 |
|  | Yes | 3542 | 27.60 |
| Education level | Before Elementary school | 5349 | 41.60 |
|  | Elementary school | 3812 | 29.70 |
|  | Middle school | 2461 | 19.20 |
|  | High school | 1056 | 8.20 |
|  | Above Three Year College | 168 | 1.30 |

N, Mean±SD; %, n/N.

**Supplementary Table 3.** **Univariable analysis in CHARLS between blood-based biomarkers and MD**

| **Characteristics** |  | **HC (%)** | **Case (%)** | **OR (95%CI)** | ***P*** |
| --- | --- | --- | --- | --- | --- |
| BG |  | 103.4 ± 34.2 | 103.2 ± 36.9 | 1.00 (1.00-1.00) | 0.760 |
| BUN |  | 15.4 ± 4.6 | 15.5 ± 4.9 | 1.00 (1.00-1.01) | 0.258 |
| CRP |  | 2.6 ± 5.6 | 3.0 ± 6.6 | 1.01 (1.01-1.02) | **<0.001** |
| CysC |  | 0.8 ± 0.2 | 0.9 ± 0.3 | 1.31 (1.11-1.53) | **0.001** |
| HbA1c |  | 6.0 ± 1.0 | 6.0 ± 1.1 | 1.03 (0.99-1.07) | 0.177 |
| HCT |  | 41.8 ± 5.7 | 40.5 ± 5.5 | 0.96 (0.95-0.97) | **<0.001** |
| HDL-C |  | 51.0 ± 11.4 | 51.7 ± 11.7 | 1.01 (1.01-1.01) | **0.002** |
| HGB |  | 13.8 ± 1.9 | 13.4 ± 1.9 | 0.89 (0.87-0.91) | **<0.001** |
| LDLc |  | 102.1 ± 28.8 | 102.5 ± 29.3 | 1.00 (1.00-1.00) | 0.402 |
| MCV |  | 91.4 ± 7.7 | 91.1 ± 8.0 | 0.99 (0.99-0.99) | **0.039** |
| PLT |  | 204.8 ± 74.4 | 205.8 ± 79.2 | 1.00 (1.00-1.00) | 0.523 |
| SCr |  | 0.8 ± 0.3 | 0.8 ± 0.3 | 0.63 (0.53-0.76) | **<0.001** |
| TCHO |  | 183.5 ± 36.2 | 184.7 ± 37.7 | 1.00 (1.00-1.00) | 0.123 |
| TG |  | 143.8 ± 91.6 | 141.5 ± 90.5 | 1.00 (1.00-1.00) | 0.218 |
| UA |  | 5.0 ± 1.4 | 4.7 ± 1.4 | 0.87 (0.84-0.89) | **<0.001** |
| WBC |  | 6.0 ± 1.8 | 6.0 ± 2.7 | 0.99 (0.96-1.01) | 0.172 |
| Age |  | 59.0 ± 10.1 | 60.0 ± 9.8 | 1.01 (1.01-1.01) | **<0.001** |
| Gender | Male | 4806 (50.4) | 1131 (34.2) | Ref |  |
|  | Female | 4730 (49.6) | 2179 (65.8) | 1.96 (1.80-2.13) | **<0.001** |
| Alcohol consumption | None | 5950 (62.4) | 2367 (71.5) | Ref |  |
|  | Yes | 3586 (37.6) | 943 (28.5) | 0.66 (0.61-0.72) | **<0.001** |
| Smoking | None | 6754 (70.8) | 2550 (77) | Ref |  |
|  | Yes | 2782 (29.2) | 760 (23) | 0.72 (0.66-0.79) | **<0.001** |
| Economic situation |  | 22125.4 ± 238014.5 | -6106.6 ± 350745.9 | 1.00 (1.00-1.00) | **<0.001** |
| Education level | Before Elementary school | 3513 (36.8) | 1836 (55.5) | Ref |  |
|  | Elementary school | 2972 (31.2) | 840 (25.4) | 0.54 (0.49-0.59) | **<0.001** |
|  | Middle school | 1978 (20.7) | 483 (14.6) | 0.47 (0.42-0.52) | **<0.001** |
|  | High school | 917 (9.6) | 139 (4.2) | 0.29 (0.24-0.35) | **<0.001** |
|  | Above Three Year College | 156 (1.6) | 12 (0.4) | 0.15 (0.08-0.27) | **<0.001** |

HC, health control; Case, depressive symptoms case; Ref, reference.

**Supplementary Table 4. MR analysis between blood-based biomarkers and MD utilizing IVW method**

| **Exposure** | **Category** | **Outcome** | **OR** | **OR_95%low** | **OR_95%up** | ***P*** | ***Padjust*** |
| --- | --- | --- | --- | --- | --- | --- | --- |
| LDLc | Metabolic | MD | 0.869 | 0.786 | 0.960 | **0.006** | **0.035** |
| BASO | Blood cell | MD | 1.172 | 1.035 | 1.326 | **0.012** | 0.160 |
| TG | Metabolic | MD | 1.127 | 0.994 | 1.278 | 0.061 | 0.183 |
| TCHO | Metabolic | MD | 0.916 | 0.813 | 1.032 | 0.148 | 0.296 |
| BG | Metabolic | MD | 0.848 | 0.642 | 1.120 | 0.246 | 0.368 |
| MCH | Blood cell | MD | 0.956 | 0.882 | 1.036 | 0.271 | 0.987 |
| WBC | Blood cell | MD | 1.070 | 0.933 | 1.228 | 0.335 | 0.987 |
| CRP | Inflammatory | MD | 0.929 | 0.754 | 1.146 | 0.492 | 0.492 |
| MCV | Blood cell | MD | 0.973 | 0.900 | 1.052 | 0.496 | 0.987 |
| MCHC | Blood cell | MD | 0.945 | 0.803 | 1.113 | 0.497 | 0.987 |
| HGB | Blood cell | MD | 0.931 | 0.741 | 1.170 | 0.540 | 0.987 |
| LYMPH | Blood cell | MD | 0.938 | 0.734 | 1.200 | 0.613 | 0.987 |
| RBC | Blood cell | MD | 1.031 | 0.916 | 1.160 | 0.616 | 0.987 |
| HbA1c | Metabolic | MD | 0.956 | 0.746 | 1.224 | 0.720 | 0.864 |
| UA | Kidney-related | MD | 0.979 | 0.842 | 1.138 | 0.786 | 0.989 |
| HDL-C | Metabolic | MD | 0.994 | 0.910 | 1.086 | 0.902 | 0.902 |
| EO | Blood cell | MD | 0.992 | 0.858 | 1.147 | 0.912 | 0.987 |
| HCT | Blood cell | MD | 1.010 | 0.819 | 1.246 | 0.926 | 0.987 |
| NEUT | Blood cell | MD | 0.994 | 0.849 | 1.163 | 0.940 | 0.987 |
| PLT | Blood cell | MD | 0.996 | 0.900 | 1.102 | 0.941 | 0.987 |
| BUN | Kidney-related | MD | 1.003 | 0.846 | 1.189 | 0.972 | 0.989 |
| MONO | Blood cell | MD | 1.001 | 0.876 | 1.144 | 0.987 | 0.987 |
| SCr | Kidney-related | MD | 1.001 | 0.826 | 1.213 | 0.989 | 0.989 |

OR, odds ratio; *Padjust*, using a false discovery rate (FDR) threshold of 5%.

**Supplementary Table 5. Univariable MR analysis** **between blood-based biomarkers and MD utilizing alternative methods**

| **Trait_pair** | **Method** | **OR** | **OR_95%low** | **OR_95%up** | **SE** | ***P*** |
| --- | --- | --- | --- | --- | --- | --- |
| BASO-MD | MR Egger | 1.116 | 0.859 | 1.450 | 0.170 | 0.418 |
| BASO-MD | Weighted median | 1.224 | 1.009 | 1.484 | 0.133 | **0.040** |
| BASO-MD | Weighted mode | 1.211 | 0.997 | 1.470 | 0.132 | 0.062 |
| BASO-MD | MR-PRESSO | 1.172 | 1.053 | 1.304 | 0.067 | **0.006** |
| BASO-MD | GSMR | 1.179 | 1.049 | 1.325 | 0.074 | **0.006** |
| BASO-MD | CAUSE | 1.073 | 0.970 | 1.185 | 0.058 | 0.490 |
| LDLc-MD | MR Egger | 0.976 | 0.852 | 1.119 | 0.073 | 0.734 |
| LDLc-MD | Weighted median | 0.953 | 0.841 | 1.080 | 0.065 | 0.455 |
| LDLc-MD | Weighted mode | 0.941 | 0.833 | 1.064 | 0.062 | 0.341 |
| LDLc-MD | MR-PRESSO | 0.869 | 0.786 | 0.960 | 0.047 | **0.011** |
| LDLc-MD | GSMR | 0.900 | 0.831 | 0.975 | 0.038 | **0.010** |
| LDLc-MD | CAUSE | 0.861 | 0.787 | 0.932 | 0.037 | **0.010** |

CAUSE, Causal Analysis Using Summary Effect Estimates; GSMR, Generalized Summary-data-based Mendelian Randomization; SE, standard error.

**Supplementary Table 6. Heterogeneity analysis of Inverse Variance Weighted between blood-based biomarkers and MD**

| **Exposure** | **Outcome** | **Q** | **Q_pval** |
| --- | --- | --- | --- |
| BASO | MD | 25.114 | 0.866 |
| BG | MD | 17.712 | 0.341 |
| BUN | MD | 35.025 | 0.732 |
| CRP | MD | 3.775 | 0.437 |
| EO | MD | 39.255 | 0.285 |
| HbA1c | MD | 31.897 | 0.102 |
| HCT | MD | 45.848 | 0.278 |
| HDL-C | MD | 52.378 | 0.181 |
| HGB | MD | 40.978 | 0.300 |
| LDLc | MD | 24.963 | 0.408 |
| LYMPH | MD | 19.419 | 0.366 |
| MCH | MD | 131.999 | 0.066 |
| MCHC | MD | 80.937 | 0.002 |
| MCV | MD | 134.311 | 0.118 |
| MONO | MD | 54.591 | 0.238 |
| NEUT | MD | 36.758 | 0.124 |
| PLT | MD | 133.257 | 0.192 |
| RBC | MD | 97.311 | 0.233 |
| SCr | MD | 90.995 | 0.065 |
| TCHO | MD | 38.626 | 0.620 |
| TG | MD | 51.549 | 0.016 |
| UA | MD | 64.447 | 0.008 |
| WBC | MD | 68.900 | 0.315 |

Q, Cochran’s Q; Q_pval, p-value of the Cochran’s Q.

**Supplementary Table 7. Intercept term from MR Egger regression and heterogeneity analysis**

| **Exposure** | **Outcome** | **Method** | **Q** | **Q_pval** | **intercept** | **int_pval** |
| --- | --- | --- | --- | --- | --- | --- |
| BASO | MD | MR Egger | 24.942 | 0.842 | 0.003 | 0.681 |
| BG | MD | MR Egger | 17.439 | 0.293 | 0.011 | 0.635 |
| BUN | MD | MR Egger | 34.356 | 0.722 | 0.007 | 0.418 |
| CRP | MD | MR Egger | 3.613 | 0.306 | -0.008 | 0.738 |
| EO | MD | MR Egger | 37.339 | 0.318 | -0.012 | 0.195 |
| HbA1c | MD | MR Egger | 30.530 | 0.106 | 0.016 | 0.332 |
| HCT | MD | MR Egger | 43.667 | 0.318 | -0.011 | 0.165 |
| HDL-C | MD | MR Egger | 51.870 | 0.166 | -0.003 | 0.520 |
| HGB | MD | MR Egger | 40.486 | 0.279 | -0.006 | 0.513 |
| LDLc | MD | MR Egger | 19.096 | 0.696 | -0.013 | 0.024 |
| LYMPH | MD | MR Egger | 17.555 | 0.417 | 0.029 | 0.197 |
| MCH | MD | MR Egger | 129.947 | 0.074 | 0.005 | 0.194 |
| MCHC | MD | MR Egger | 80.554 | 0.002 | -0.004 | 0.639 |
| MCV | MD | MR Egger | 125.041 | 0.246 | 0.010 | 0.004 |
| MONO | MD | MR Egger | 53.830 | 0.229 | 0.007 | 0.419 |
| NEUT | MD | MR Egger | 36.749 | 0.100 | -0.001 | 0.938 |
| PLT | MD | MR Egger | 129.393 | 0.243 | 0.008 | 0.062 |
| RBC | MD | MR Egger | 96.470 | 0.229 | -0.004 | 0.386 |
| SCr | MD | MR Egger | 86.443 | 0.103 | 0.014 | 0.057 |
| TCHO | MD | MR Egger | 37.427 | 0.630 | -0.005 | 0.280 |
| TG | MD | MR Egger | 51.498 | 0.012 | -0.001 | 0.862 |
| UA | MD | MR Egger | 59.673 | 0.018 | 0.011 | 0.085 |
| WBC | MD | MR Egger | 68.523 | 0.296 | 0.004 | 0.558 |

Q, Cochran’s Q; Q_pval, p-value of the Cochran’s Q; int_pval, p-value of the intercept.
